# Chitosan/Essential Oils Formulations for Potential Use as Wound Dressing: Physical and Antimicrobial Properties

**DOI:** 10.3390/ma12142223

**Published:** 2019-07-10

**Authors:** Elaine Pereira dos Santos, Pedro Henrique Medeiros Nicácio, Francivandi Coêlho Barbosa, Henrique Nunes da Silva, André Luís Simões Andrade, Marcus Vinícius Lia Fook, Suédina Maria de Lima Silva, Itamara Farias Leite

**Affiliations:** 1 Materials Engineering Department, Federal University of Paraíba, João Pessoa PB 58051-900, Brazil; 2 Materials Engineering Department, Federal University of Campina Grande, Campina Grande PB 58429-140, Brazil

**Keywords:** chitosan, clove, melaleuca, films, antimicrobial activity, physical properties

## Abstract

Film-forming emulsions and films, prepared by incorporating different concentrations of clove essential oil (CEO) and melaleuca essential oil (MEO) into chitosan (CS) were obtained and their properties were evaluated. Film-forming emulsions were characterized in terms of qualitative assessment, hydrogen potential and in vitro antibacterial activity, that was carried by the agar diffusion method, and the growth inhibition effects were tested on the Gram-positive microorganism of *Staphylococcus aureus*, Gram-negative microorganisms of *Escherichia coli*, and against isolated fungi such as *Candida albicans*. In order to study the impact of the incorporation of CEO and MEO into the CS matrix, the appearance and thickness of the films were evaluated. Furthermore, Fourier transform infrared spectroscopy (FTIR), contact angle measurements, a swelling test, scanning electron microscopy and a tensile test were carried out. Results showed that the film-forming emulsions had translucent aspect with cloudy milky appearance and showed antimicrobial properties. The CEO had the highest inhibition against the three strains studied. As regards the films’ properties, the coloration of the films was affected by the type and concentration of bioactive used. The chitosan/CEO films showed an intense yellowish coloration while the chitosan/MEO films presented a slightly yellowish coloration, but in general, all chitosan/EOs films presented good transparency in visible light besides flexibility, mechanical resistance when touched, smaller thicknesses than the dermis and higher wettability than chitosan films, in both distilled water and phosphate-buffered saline (PBS). The interactions between the chitosan and EOs were confirmed by. The chitosan/EOs films presented morphologies with rough appearance and with EOs droplets in varying shapes and sizes, well distributed along the surface of the films, and the tensile properties were compatible to be applied as wound dressings. These results revealed that the CEO and MEO have a good potential to be incorporated into chitosan to make films for wound-healing applications.

## 1. Introduction

Essential oils are volatile liquids, characterized by a strong odor, extracted from plants or spices. Their commercial production is mainly by the steam distillation method. However, fermentation, expression or solvent extraction processes are also used [1]. The interest in essential oils for application in various industrial segments, such as food, perfume, aromatherapy and pharmaceuticals, is due their potential benefits [2,3]. Besides antibacterial and antifungal activities, essential oils have antiviral, insecticidal, anti-tumour, analgesic, anti-diabetic, anti-inflammatory and antioxidant properties [4,5,6,7,8,9,10,11]. According to literature, the antimicrobial effects of essential oils are coming mostly from polyphenols and terpenes, namely monoterpenes, eugenol, cinnamaldehyde, carvacrol, and thymol [2,12,13,14,15,16,17,18,19]. Thus, the composition, structure and functional groups of the oils determine their antimicrobial activity [20].

Of the many essential oils, melaleuca oil and clove oil are known for their high therapeutic potential. The essential oil of *Melaleuca alternifolia*, also named as tea tree oil (TTO), is a complex mixture of terpen hydrocarbons and tertiary alcohols [21]. This oil has been used successfully in the handling of oral candidosis in (acquired immunodeficiency syndrome) AIDS patients [22] and other oral fungal infections in patients suffering from advanced cancer [23]. This is due the fact that the TTO represents a good alternative to the most commonly used antifungal drugs, because yeasts often show resistance to them [23,24]. The clove oil (*Syzygium aromaticum*) is a natural essential oil with antimicrobial and antioxidant activities, containing active compounds including eugenol, eugenol acetate, and β- caryophyllene [25,26], which are responsible for anti-oxidant activity [27,28], anti-stress [29], and anti-microbial agent [2,30,31,32,33,34,35,36,37] properties of the oil. Clove oil can be used by several industries for different purposes, like food, health, and personal care products manufacture.

Melaleuca and clove oils have been intensively investigated due to their promising advantages to human health. However, the compositions and quality of the ones are influenced by the plant characteristics, extraction method, analysis conditions and the solvent used. [38,39,40,41]. Thus, the present work had the purpose of characterizing the melaleuca and clove essential oils, both provided by Mundo Verde (Rio de Janeiro, Brazil), and assessment of their antimicrobial properties against Gram-positive microorganism of *Staphylococcus aureus*, Gram-negative microorganisms of *Escherichia coli*, and against isolated fungi as *Candida albicans*. Moreover, since these oils are unstable volatile compounds which can be degraded easily (by oxidation, volatilization, heating, light, etc.) when they are used in the free form, they were encapsulated in chitosan as a useful alternative to enhance their stability and to increase their action duration. According to literature, encapsulating clove oil or eugenol using nanoparticles or nanoemulsions increases the stability of the compounds [42,43,44]. According to literature, encapsulating clove oil and salvia oil using nanoliposomes improves the stability of the oils prior to administration [36,45].

Various essential oils have been encapsulated into chitosan [46,47,48,49,50,51,52]. The emulsification technique followed by solidification is generally used to the encapsulation [42,49,53]. The incorporation of essential oils into chitosan aiming at the manufacture of films for therapeutic use, like wound dressings, represents an important area of research. The reason for the close attention in the use of chitosan, which is an Food and Drug Administration (FDA) approved biopolymer, as a potent material for future wound dressing is because this biopolymer influence positively into diverse steps of involved wound healing process [54,55,56]. Chitosan is pointed out for treatment of wounds due to its beneficial biological properties, specifically hemostatic action, hydrating effect, anti-inflammatory characteristics, and angiogenic activity. It accelerates dermal regeneration and re-epithelization of skin promoting wound healing [57,58,59]. In addition, chitosan has been proved to be non-toxic, non-immunogenic, non-carcinogenic, biodegradable, biocompatible, bioadherent and blocks nerve endings to reduce pain [60,61,62,63,64]. Despite the benefits that can be achieved with the chitosan and essential oils association for medicinal purposes, research concerning the use of chitosan films for wound dressing is still limited [55,65,66,67,68,69,70]. Thus, the aim of this study was identify if the chitosan, from shrimp shells, prepared in our laboratory—Northeastern Biomaterials Evaluation and Development Laboratory (CERTBIO, Campina Grande, PB, Brazil)—can be used as a matrix for encapsulating melaleuca and clove essential oils in order to produce chitosan/essential oils formulations for potential use as wound dressing. Films, prepared by incorporating different concentrations of melaleuca and clove essential oils into chitosan, were obtained and the impact of the incorporation of these oils into the physical, optical and structural properties of the resulting films was evaluated.

## 2. Materials and Methods

### 2.1. Materials

Chitosan (CS), from shrimp shells, medium molecular weight, 230–250 kDa determined by viscosimetry [71] and degree of deacetylation (DD) of ca. 85% determined by the infrared spectroscopy method [72], containing an insoluble residue content of less than 1%, was prepared in CERTBIO. Lactic acid (Purity 85%) was purchased from Neon (São Paulo, Brazil). Clove essential oil (CEO) (Eugenia caryophyllata or Syzygium aromaticum) and melaleuca essential oil (MEO) (Melaleuca alternifolia), 100% pure and natural of Phytotherapica, both provided by Mundo Verde (Rio de Janeiro, Brazil) and used as received. These oils are colorless and with strong and characteristic odors. Distilled water was used in all experiments.

### 2.2. Chitosan/Essential Oils Film Preparation

Chitosan (0.7 g) was dissolved in 70 mL of 1% (*v*/*v*) lactic acid solution under continuous stirring for 2 h at 45 ± 5 °C. After this period, the chitosan solution (CS-S) obtained was poured into Petri dishes, dried in an oven at 40 °C for 30 h to evaporate the solvent and to obtain the chitosan films (CS-F). For chitosan/essential oils emulsions preparation, the chitosan solution was mixed with 1% and 3% (*v*/*v*) of CEO and MEO under magnetic stirring for more 3 h at 45 ± 5 °C. The emulsions were coded as CS/1CEO-E, CS/3CEO-E, CS/1MEO-E and CS/3MEO-E for those prepared with 1% and 3% of clove and melaleuca essential oils, respectively. These emulsions were poured on Petri dishes and dried in the same condition described above aiming to produce the chitosan/essential oil films. The chitosan/essential oil films were peeled off gently, placed on parchment paper and covered with aluminum foil to protect from light. The films with 1% and 3% of clove and melaleuca essential oils were labeled as CS/1CEO-F, CS/3CEO-F, CS/1MEO-F and CS/3MEO-F, respectively.

### 2.3. Characterization

#### 2.3.1. Qualitative Assessment

The chitosan solutions and chitosan/essential oils emulsions were qualitatively analyzed for solubility, viscosity, color, presence of bubbles, and their films for color, presence of impurities, flaws, adhesion, flexibility and mechanical resistance, using visual analysis and touch. The films’ thickness measurements were assessed using an average of 6 points distributed throughout the film and analyzed statistically. These measures were carried out with a Mitutoyo Europe GmbH micrometer (Neuss, Germany) which has a measurement capacity of 0–25 mm, a 0.001 mm resolution.

#### 2.3.2. Antimicrobial Susceptibility

Antimicrobial activity of essential oils, chitosan solution and chitosan/essential oils emulsions were investigated using the dish diffusion method reported by Pelissari, et al. [73], with some modifications. The samples were handled in a sterile environment using Quimis model Q216F21RA1 biological safety booth. For the assay was used culture medium Mueller Hinton (MH) (Kasvi-Italy broth) added with solidifying medium (Bacto Agar). The strains employed in this study were the Gram-positive bacteria *Staphylococcus aureus* (ATCC 25923) and gram-negative *Escherichia coli* (ATCC 25922), and the yeast *Candida albicans* (ATCC 10231). All the strains used had a 24 h growth. Firstly, a suspension was prepared in a concentration of 0.5 on Mcfarlad scale, using 0.9% sodium chloride for bacteria and distilled water for yeast. Then, with the aid of a suabe, the suspension was spread on MH agar plates; 30 μL of freshly prepared compound was loaded on 5 mm diameter filter paper discs and then placed on the MH agar plates with the inoculum. The plates were incubated in a bacteriological heater at 35 °C for bacteria and 37 °C for yeast, both for 48 h with the measure of the diameter of the inhibition zone every 24 h. The results were measured by the mean of the two zone of inhibition measured (24 h and 48 h) without subtracting the diameter of the discs.

#### 2.3.3. Fourier Transform Infrared Spectroscopy (FTIR)

The spectra in the infrared region of the chitosan and chitosan/essential oils films were recorded at room temperature in a Spectrum 400 spectrophotometer from PerkinElmer (Waltham, MA, USA), coupled to the Attenuated Total Reflection (ATR) module, diamond crystal and ZnSe prism in the spectral region of 4000 to 600 cm^−1^, with a resolution of 4 cm^−1^ and 64 scans.

#### 2.3.4. Wettability

Static contact angles of the films were measured using a contact angle goniometry (Ramé-Hart, model 190 CA, CERTBIO—Campina Grande, PB, Brazil). Distilled water (50 µL) at pH 5.1 and phosphate buffer (pH 7.2) were dropped onto the surface of the films before measuring. The contact angle data was obtained from the images of water droplets on film surface.

#### 2.3.5. Swelling Degree

Films samples of known mass (M_0_) were immersed in distilled water (pH 5.5) and phosphate buffer solution (pH 6.4) at ambient temperature until swelling equilibrium was attained for periods from 1, 2 and 3 h. The mass of swollen sample (M_s_) was measured after removing the surface water with filter paper. Swelling degree (SD) was then calculated from Equation (1). The assay was performed in triplicate and followed the methodology of Pal and Pal [74] with some adaptations.

(1) $$\mathrm{SD}(\%)=\frac{M_{s}-M_{0}}{M_{0}}\times 100$$

where:

M_0_—film mass before swelling (g)
M_s_—film mass after swelling (g)

#### 2.3.6. Scanning Electron Microscope (SEM)

Surface morphology of all the films was analyzed using scanning electron microscopy (SEM) with the RG Zeiss, model LEO 1430 (Zeiss, Oberkochen, Germany). A fine layer of gold (22.5 nm) was deposited over the surface prior to test using Emitech, model K550X sputter coater (Kent, UK). The surface images were generated using a secondary electron detector under vacuum and voltage of 10 kV.

#### 2.3.7. Tensile Properties

Tensile tests were performed according to ASTM D 882. The properties were measured on a Shimadzu Universal Tester (Kyoto, Japan), Model autograph AG-X 10 KN. The parameters adopted were: load cell of 20 Kgf, speed of 5 mm/min and rectangular sample with 10 mm width, base length of 50 mm and thickness ranging from 0.049 to 0.173 mm, according to with the analyzed composition. Average value of six samples was reported for each composition. All of the measurements were carried out at ambient temperature. This assay was performed to evaluate tensile strength (TS), elongation at break (EB) and Young’s modulus (MY) of the films.

#### 2.3.8. Statistical Analysis

Hydrogen potential of chitosan solution and chitosan/essential oils emulsions, as well as, the thickness, wettability and swelling values of the chitosan and chitosan/essential oils films were expressed as mean ± standard deviation. The data were evaluated using analysis of variance (ANOVA), and the significance of the model was verified with Tukey’s test, with a significance level of 95% (*p* < 0.05) using a statistical analysis and design of experiments program, Sisvar (5.6 Version, register number: 828459851, Lavras, MG, Brazil) [75].

## 3. Results and Discussion

### 3.1. Qualitative Assessment of the Chitosan Solution and Chitosan/Essential Oils Emulsions

**Figure 1:**
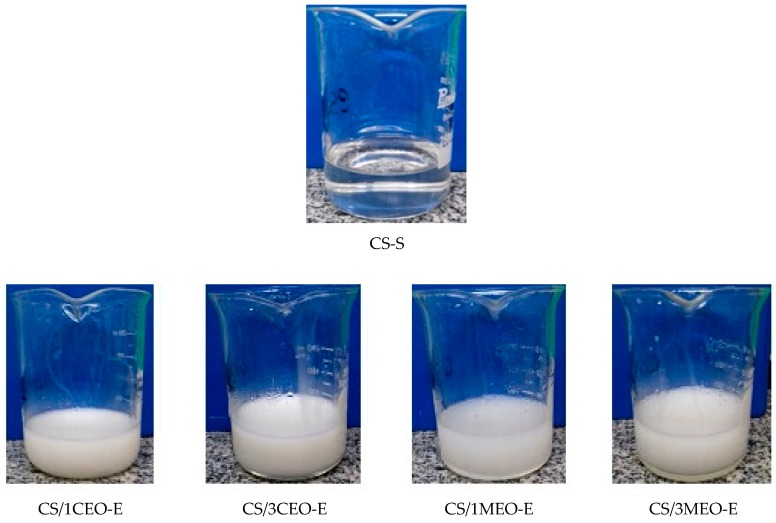
Images of chitosan solution and chitosan/clove and melaleuca essential oils emulsions.

The appearance of the chitosan solution (CS-S) and chitosan/clove and melaleuca essential oils emulsions (CS/1CEO-E, CS/3CEO-E, CS/1MEO-E, and CS/3MEO-E) are shown in Figure 1. The chitosan used in this study, with an impurities content of less than 1%, presented good solubility in lactic acid and an excellent optical property of transparency in visible light (colorless) and the chitosan/essential oils emulsions had translucent aspect with cloudy milky appearance, suggesting that droplet radius is possibly higher than to 100 nm. According to Mason, et al. [76], the nanoemulsions presented visual aspect different from macroemulsions. This behavior is due to the radius of the droplets being much smaller than the wavelength in the visible range, presenting optical transparency for this case. On the other hand, nanoemulsions with droplets of approximately 100 nm, have a hazy appearance and, above this value, the nanoemulsions have a milky appearance (white) due to the multiple scattering of the light beam in the visible region. In this case, it is suggested that both the chitosan molecules and the oil droplets, due to their lack of affinity, showed phase separation in particular, when a volume from 3% from oil was used in the formulations [77,78,79].

A particularity to be highlighted in the prepared emulsions is the strong and characteristic odor of each oil. CEO has a penetrating and warm aroma, while the herbaceous aroma prevails over MEO.

### 3.2. Hydrogen Potential (pH) of the Chitosan Solution and Chitosan/Essential Oils Emulsions

The physicochemical properties of chitosan in solution are significantly influenced by the pH of the medium. In acid solution, the amino groups present in the chitosan chain are protonated, limiting the interactions between macromolecules due to electrostatic repulsion [80]. At higher pH than the pKa of 6.3, chitosan’s amines become deprotonated, and favor the formation of hydrogen bonds between the hydroxyl groups and the amine groups of chitosan [81]. Thus, the polymer loses its charge and becomes insoluble with gel or film forming characteristics. Since the lactic acid solution at 1% *v/v* presented pH 1.8 ± 0.01, the amino groups of the chitosan easily become protonated, favoring an excellent solubility of the biopolymer during the process of preparation of the polymer solutions. As reported by Luo, et al. [82] the total charge of the chitosan depends on the pH value of the medium, and consequently on the protonation or deprotonation of the amino groups present in the chitosan [83].

According to statistical data (Table 1), the hydrogen potential in all compositions studied was acid (from 2.58 to 2.62 A) and the essential oil type as well as their quantity did not affect this property (*p* < 0.05). Therefore, the chitosan solubility during the process of polymer preparation solutions was excellent. In addition, it is possible that the all emulsions to remain bactericidal, once the antibacterial activity of chitosan, in general, is enhanced under acidic condition. This is due to the presence of a large majority of positively charged amino groups as well as the exceptional solubility of chitosan [84,85,86,87]. As reported by Kong et al. [83], the chitosan molecules protonated groups favor the interaction with the negatively charged cell surface and leads to the damage of the microbial cell, resulting in the antibacterial activities.

### 3.3. In Vitro Antibacterial Activity of the Chitosan Solution and Chitosan/Essential Oils Emulsions

Natural bioactives (CEO and MEO), chitosan solution (CS-S) and chitosan/essential oils emulsions (CS/1CEO-E, CS/3CEO-E, CS/1MEO-E, and CS/3MEO-E) were evaluated by the agar diffusion method. Table 2 presents the values of the inhibition halos exhibited by each of the samples against *Staphylococcus aureus* and *Escherichia coli* bacteria, and the fungi *Candida albicans*, which according to literature [88,89,90,91,92] are the most commonly found in wounds.

Comparing the antimicrobial activity of the clove essential oil (CEO) and melaleuca essential oil (MEO), it was shown that the CEO presented the greatest inhibition against the microorganisms employed, followed by the MEO. The present study is also in agreement with that reported by Pinto, et al. [93] on the activity of CEO against isolated fungi as *Candida albicans*, whose halo was of 21 mm.

The inhibition halos observed for MEO against *Staphylococcus aureus*, *Escherichia coli* and *Candida albicans* were 9.0, 10.0 and 12.0 mm, respectively (Table 2). The fungi showed greater sensitivity to MEO, while Gram-positive bacteria showed lower sensitivity. According to Cimanga, et al. [94], essential oils that result in growth inhibition halos below 10 mm are classified as inactive, and active if they result in halos between 10 and 15 mm and very active to values above 15 mm. In this way, CEO is classified as very active for the three microorganisms and MEO as inactive for *Staphylococcus aureus* and active for *Escherichia coli* and *Candida albicans*.

Chitosan presents an intrinsic antimicrobial activity. The chitosan solution (CS-S) showed activity against the 3 strains used in this study as observed in Table 2. The antimicrobial activity of chitosan can be attributed to the binding of the positive charges present on the chitosan with the negative charges existing on the surface of the bacteria through electrostatic interactions that can cause structural damage, leading to cell death [87,95,96,97]. In addition, antimicrobial activity can also be governed by molecular weight, degree of deacetylation, solubility, pH of solution, temperature, viscosity as well as by the composition of the bacterial cell walls [96]. Mohamed and El-Ghany [98] and Helander, et al. [99] reported the inhibitory effect of chitosan against Gram-positive and Gram-negative bacteria and fungi. In our work, the inhibition halo values for CS-S against *Staphylococcus aureus* and *Escherichia coli* and *Candida albicans* were 8.5, 7.0 and 7.5, respectively, suggesting that chitosan may be employed in the wounds treatment against these Gram-positive and Gram-negative bacteria and against fungi.

Huang, et al. [100] and Wang, et al. [101] showed that the combination of the chitosan with other compounds improve antibacterial activity by the synergistic effect of the mixture as acetate with silver nanoparticles.

According to Table 2, all compositions studied here presented antimicrobial activity in different action spectra. The compositions that showed activity against the three strains were CS-S, CS/3CEO-E and CS/1MEO-E. The incorporation of natural bioactives into the chitosan provided the different compositions’ antimicrobial activity in the emulsions in study. Among the investigated compositions, those that had more activities against Gram-positive *Staphylococcus aureus* bacteria was CS/3CEO-E; against *Escherichia coli* were: CS/3CEO-E, CS/1MEO-E, CS/3MEO-E and against the *Candida albicans* fungi were: CS/1CEO-E and CS/3MEO-E. This shows the relevance in combining the chitosan and active materials. So, chitosan emulsions prepared in this study showed discrete antimicrobial activity when compared to the essential oils (CEO and MEO). Many factors may have contributed to these results such as the formation of bonds in the polymeric medium that reduced the intense biological activity of the oils, the emulsion pH, as well as the low concentrations of bioactives incorporated. As numerous factors are involved in the healing process, in addition to the antimicrobial activity, it is valid to evaluate the behavior of chitosan emulsions with natural bioactives using other analyzes, such as healing activity in vivo.

### 3.4. Qualitative Assessment of the Chitosan and Chitosan/Essential Oils Films

**Figure 2:**
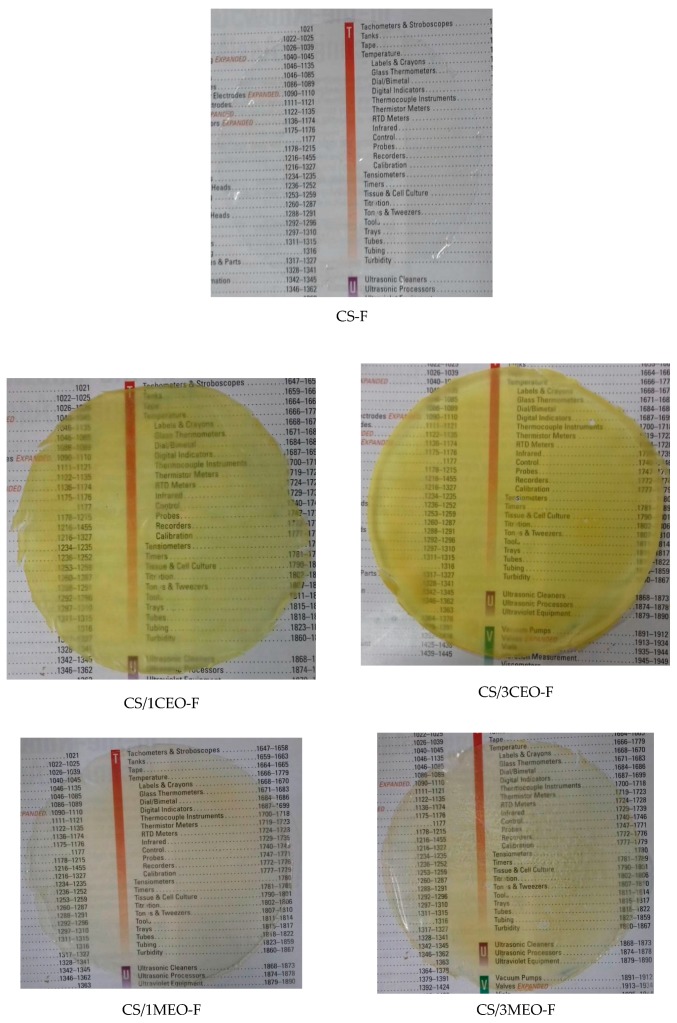
Images of the chitosan and chitosan/essential oils films.

The appearance of the chitosan and chitosan/essential oils films is shown in Figure 2. The coloration of the films was affected by the type and concentration of bioactives used. In general, all chitosan films presented good transparency in visible light besides flexibility and mechanical resistance when touched.

The chitosan/clove essential oil films (CS/1CEO-F and CS/3CEO-F) showed an intense yellowish coloration while the chitosan/melaleuca essential oil films (CS/1MEO-F and CS/3MEO-F) presented a slightly yellowish coloration. In addition, they are much more transparent to visible light than those with CEO (Figure 2). The essential oils altered the coloration of the chitosan solution as can be observed in the Figure 1. Therefore, the differences observed macroscopically in the films are consequences of the particularities in the chitosan emulsions formed by each composition.

In summary, all the films investigated were transparent, varying from colorless to yellowish, since the natural bioactive ones diminished, but did not totally extinguish the optical transparency to the visible light characteristic of the chitosan film in the study. Moreover, all chitosan and chitosan/essential oils films presented malleability, elasticity and adhesion; nevertheless, the films incorporated with the oils were shown to be less adherent and flexible than the chitosan films as qualitative analysis characteristic of the films shows in Table 3. According to these results, the produced films may be potential candidates for the treatment of wounds.

The thickness measurements of the chitosan and chitosan/essential oils films are shown in Table 4. As seen in this table, the statistical data showed that type and the concentration of the incorporated bioactives not influenced in the values of thickness of chitosan films. These films presented thickness compatible with the thickness of the epidermis [102]. Skin is the largest organ of the human body and presents well-defined anatomical regions. Among these regions, there is the epidermis that has layers with different depths depending on the region of the body and present thickness varying from 0.07 mm to 0.12 mm over much of the body surface [103,104].

### 3.5. Fourier Transform Infrared Spectroscopy (FTIR) of the Chitosan and Chitosan/Essential Oils Films

**Figure 3:**
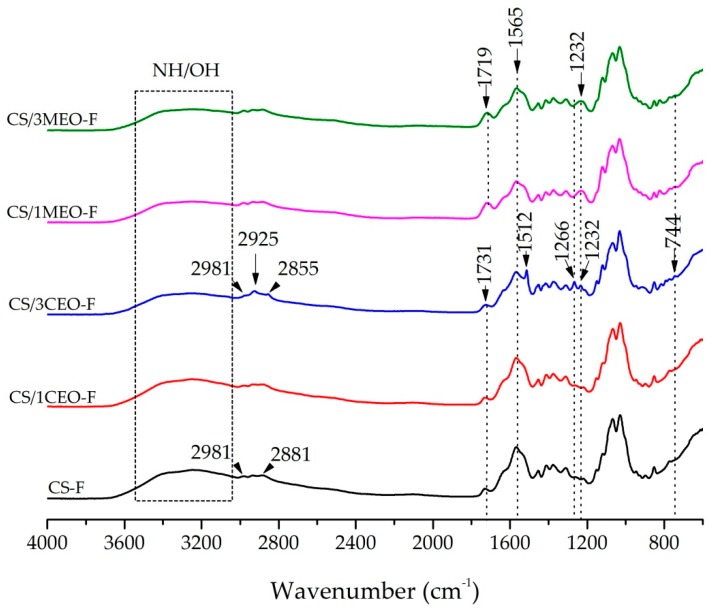
Fourier transform infrared spectroscopy (FTIR) spectra of the chitosan and chitosan/essential oils films.

The FTIR spectra of the chitosan and chitosan/essential oils films are shown in Figure 3. This characterization was conducted to determine functional group interactions between the chitosan and EOs.

No changes were observed in the FTIR spectrum of chitosan when 1% CEO was added to the film (CS/1CEO-F), showing absorption bands similar to the pure chitosan film spectrum (CS-F). The chitosan film (CS-F) spectrum shows a broad band between 3660 and 3026 cm^−1^, corresponding to the stretching vibrations of the -OH group, superimposed on the N-H band, indicating intermolecular bond formation. Bands at 2981 and 2881 cm^−1^ were associated to C–H group asymmetric and symmetric stretching vibrations, respectively [105]. In addition, they showed characteristic absorption bands at 1731 cm^−1^, belonging to axial deformation of the C=O group of the chitin, at 1640 cm^−1^ associated to the amide I group and NH_3_^+^ asymmetric deformation, and at 1565 cm^−1^ related to the amide II group, N–H bending vibration and the NH_3_^+^ symmetric deformation. This last band is related the vibrations of the protonated amine group δ (NH_3_^+^). From these results, it can be observed that the chitosan was not totally deacetylated and, for this reason, the presence of both acetylated (R–C=O) and deacetylated (-NH) groups are found in the chitosan films under study. In addition, bands in the range from 1405 and 1307 cm^−1^ were attributed to the methylene and methyl groups, between 1149 and 1029 cm^−1^, were related to the C–O group stretching from glycosidic bonds and the band at 848 cm^−1^ represent of the saccharine ring [105,106,107,108,109].

**Figure 4:**
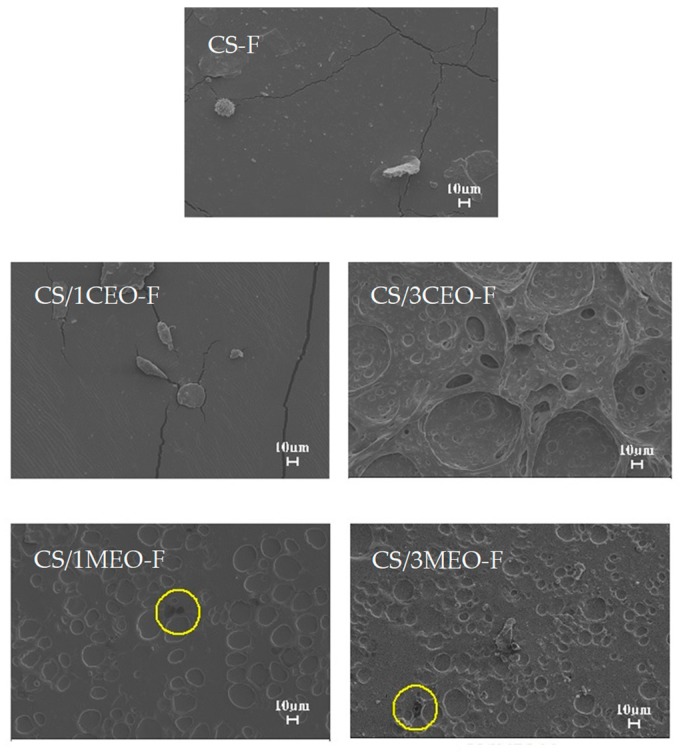
Scanning electron microscope (SEM) micrographs of the chitosan and chitosan/essential oils films.

By contrast with the CS/1CEO-F, new absorption bands were found in the chitosan spectrum when 3% of CEO was added to the film (CS/3CEO-F). Bands at 1512 cm^−1^, 1266 cm^−1^, 1232 cm^−1^, and 744 cm^−1^ were shown in spectrum CS/3CEO-F (Figure 4) related to the main constituent present in clove oil, eugenol. The characteristic bands of eugenol occurred at 1512 cm^−1^, associated to the axial deformation of C=C group of the aromatic ring; at 1266 cm^−1^ and at 1232 cm^−1^ refer to the axial stretching C-O; and the bands between 911 and 744 cm^−1^ confirm the angular deformation outside the plane of C-H group of aromatic rings [110]. It is also observed the presence of absorption bands in the range of 2981 to 2855 cm^−1^ with a new band occurring in 2925 cm^−1^, characteristics of the asymmetric and symmetrical vibrations of the CH_2_ group present in CEO oil, respectively. Absorption band displacement from 2881 cm^−1^ (CS-F) to 2855 cm^−1^ (CF/3CEO-F) was evidenced as a result of the interaction between the hydrophobic groups present in the chitosan/clove oil [111]. The CS/3CEO-F spectrum was similar to that of the eugenol standard reported by Monteiro, et al. [112], since eugenol is the major component of CEO. Then, once the infrared spectrum of the CS/3CEO-F show the presence of specific bands of CS and CEO, it can be established that there was interaction of chitosan groups with the CEO in the formation of the film, especially when 3% content was added to the film.

In the spectra of the chitosan/melaleuca oil films, CS/1MEO-F and CS/3MEO-F, (Figure 3), an increase in the band intensity at 1719 cm^−1^ was evident, characteristic of carbonyl groups C=O, also present in the MEO oil structure. This band was shifted to a low wavenumber from 1731 (CS-F) to 1719 cm^−^^1^ (CS/1MEO-F and CS/3MEO-F) with the addition of MEO. This result indicates the compatibility of the two components, as well as their interaction. The band at 1565 cm^−1^, related to the vibrations of the protonated amine group decreased with the incorporation of the oil. In addition, new band at 1232 cm^−1^, corresponding to the vibrations of the C-O bonds was observed in the spectra of the CS/1MEO-F and CS/3MEO-F and be present in the compound 1,8 cineol existent in the MEO [113]. The absence of more prominent bands characteristic of melaleuca oil may be related to the overlap of these bands with the characteristics bands of chitosan and possibly with the amount of melaleuca oil used.

### 3.6. Contact Angle Measurements of the Chitosan and Chitosan/Essential Oils Films

Contact angle values of the chitosan and chitosan/essential oils films in distilled water (pH = 5.2) and PBS (pH = 7.2) are shown in Table 5. The contact angles for the chitosan film (CS-F) in distilled water and phosphate-buffered saline (PBS) were near 64° and 68°, respectively, being classified as hydrophilic. The hydrophilicity of chitosan is attributed to the hydroxyl and amino groups present in its structure [114]. The positive charges that arise when the amino groups are protonated decrease the free energy of the surface, improving the wettability of the films [115].

According to literature, the contact angle values of chitosan with water are quite dispersed. Hsu, et al. [116], Tsai and Wang [114] found values close to 80° and Zheng, Wei, Wang, Ao, Gong and Zhang [115] reported value close to 70°. This discrepancy in the results should be associated with the difficulty of measuring the contact angle at the exact moment when the liquid drops in contact with the surface of the film and by the rapid absorption of the water by the same. Factors such as degree of deacetylation of chitosan and pH of water may also affect this measurement.

In general, it can be observed that the type and content of essential oil incorporated in the chitosan films did not influence the hydrophilicity of chitosan films in both media analyzed according to data reported in Table 5. Such behavior was confirmed by the statistical analysis.

Contact angle measurement provides important information about the character of hydrophilicity or hydrophobicity of a surface. Knowing this information about the films used as curatives can identify their behavior in response to a superficial contact with the exudate coming from the lesion. The results of hydrophilicity found in this study demonstrate that the films may be suitable for use in burns, since they promotes high humidity when in contact with the wound exudate, characterizing, therefore, an essential requirement when it comes to the elaboration of films for use as dressings [117].

### 3.7. Swelling Test of the Chitosan and Chitosan/Essential Oils Films

In the chemical structure of chitosan, there are predominant amino groups characterized by electronegative covalent bonds (N–H), which generate sites of high polarity that end up favoring the rearrangement of the water molecules around them. Associated with this characteristic, the acetamido groups also present in the polymer makes it a material with a high degree of affinity and water retention. This ability to absorb and retain water is an important factor in implantable materials, since it allows the absorption of body fluids and the transfer of nutrients and metabolites [118].

The study of the swelling parameters of polymeric systems for the mechanistic understanding of the diffusion process of the solvent into the polymers is fundamental in curative materials [119]. The swelling characteristics of chitosan and chitosan/essential oils films were analyzed by parameters associated with the amount of distilled water and PBS solution absorbed by the polymer matrix as a function of the time from 1, 2 and 3 h. The results of this absorption are in Table 6. Both in distilled water and in PBS, the CS-F completely solubilized in these media in less than 1 h of assay. This is, possibly, due to the low pH value (2.6) obtained in the chitosan solution, due to the presence of a large number of protonated groups. In addition, an excess of residual H^+^, thus generating great electronic repulsion when in contact with the fluids, with consequences undesirable as the reduction of its structural stability in the presence of moisture, leading to the disintegration of its fibers [80,81].

Kim, et al. [120] have shown that the application of chitosan films is limited in guided tissue regeneration (GTR), due to their high affinity for water. Thus, the results of the swelling test for the CS-F suggest the need for the incorporation of materials that improve their absorption rates and their stability.

The incorporation of CEO and MEO into the chitosan films (CS-F) promoted discrete changes in the uptake of fluids in the swelling test as shown by the statistical data displayed in Table 6.

The chitosan films incorporated with 1% CEO (CS/1CEO-F) showed no changes in swelling values during the three hours of analysis in distilled water. In PBS, this swelling value was higher for 1 h, while for the other periods under study a similar degree of swelling was observed. The chitosan film incorporated with 3% CEO (CS/3CEO-F) was unstable in the presence of distilled water, disintegrating in 1h assay. In PBS, this sample had similar absorption rate at the 3 assay times in relation to the CS/1CEO-F.

When 1% MEO was incorporated, an increase in the degree of swelling for CS/1MEO-F was observed in the 3 h of the test in distilled water in relation to samples containing 3% MEO (CS/3MEO-F). When comparing the same sample (CS/1MEO-F) with that of the PBS fluid, a greater swelling was observed in the period of 1 h, remaining constant in the other periods of the test. On the other hand, when analyzing the degree of swelling of samples containing MEO in PBS, a greater degree of swelling was observed for the samples containing 1% MEO (CS/1MEO-F) when compared to samples containing 3% MEO (CS/3MEO-F) according to the statistical analysis shown in Table 6. Similar behavior was observed for samples containing MEO in distilled water.

Films produced in this work have high hydrophilicity that can become gels upon contact with biological fluids, allowing high humidity in the wound region, one of the essential requirements for use as an ideal dressing for wound treatment [121].

The swelling behavior is decisive for adequate diffusion of nutrients and cells. A higher degree of swelling represents a greater ability to absorb exudates from the surface of cutaneous wounds, a factor that is very relevant in the healing process [122].

### 3.8. Scanning Electron Microscopy (SEM) of the Chitosan and Chitosan/Essential Oils Films

Figure 4 shows the surface morphology of the chitosan and chitosan/essential oils films. The CS-F presents a smooth surface, but with the presence of punctual agglomerates and fissures. The latter, possibly attributed to the lactic acid used in the preparation of the polymer solution or to the proper detachment of the petri dish sample or even the drying conditions of the material [123,124].

The incorporation of CEO at 1 and 3% in the CS film resulted in differentiated morphologies as observed in Figure 4. The CS/1CEO-F presented a surface like that of the CS-F, but with the presence of large agglomerates and cracks along the surface, showing some mechanical fragility due to the characteristic immiscibility of the components of the mixture, CS/CEO.

The surface of the CS/3CEO-F was shown to be rough and irregular, also presenting voids with different shapes, spongy appearance as a possible result of the formation of CEO droplets with varied diameters that were heterogeneously distributed throughout the chitosan film. Similar results were observed by Sánchez-González, et al. [125] when tea tree essential oil was added to the hydroxyl propyl methyl cellulose film.

The films CS/1MEO-F and CS/3MEO-F presented morphologies with rough appearance and presence of MEO droplets with varying shapes and sizes, well distributed along the surface of the films. Dark spots marked by yellow circles were observed in the films but are clearer in the SEM images of the CS/1MEO-F. Dark spots may be indicative of oily material deposited in the film. In this work, the dark spots represent the oil that did not solubilize in the polymer solution.

### 3.9. Tensile Test of the Chitosan and Chitosan/Essential Oils Films

The mechanical strength of materials elaborated for use as dressings is one of the essential requirements for the evaluation of their performance for topical use in the treatment of wounds. Depending on the application, the biodegradable films must withstand the mechanical loads required during their operation, transport and handling in order to maintain their integrity and diverse properties, such as barrier, tensile strength and deformation before breaking [126].

In addition, these materials should be flexible, and present mechanical resistance sufficient to be manipulated and transported during all the wound treatment [127]. In order to evaluate the mechanical properties of pure chitosan membranes and incorporated with bioactives, the tensile test was performed to determine the properties of maximum tensile strength, elongation at break and Young’s modulus, shown in Table 7.

The films presented a behavior like that of elastomers, with high elongation values and continuous increase of the tensile strength. The CS-F showed maximum stress of 20.3 MPa and elongation at break of 43.0%. This behavior can be attributed to the fact that the lactic acid used in this study tends to form membranes with greater flexibility and adhesion, presenting lower tensile strength, considering that it can also act as a plasticizer, giving greater mobility and flexibility in the polymer chain. While acetic acid forms more rigid films with greater tensile strength, since after the preparation of the films, they are drier and with greater restriction of movement.

Young’s modulus value of 57.4 MPa for chitosan films, higher than that found in this work (35.1 MPa), was reported by Yousefi, et al. [128] in their studies on chitosan nanofibers/henna extract using acetic acid as solvent. Not only the solvent, but many other factors can influence the mechanical properties of the films as degree of deacetylation of chitosan, solution pH, presence of plasticizer and mixing process [129,130,131].

The incorporation of the natural bioactives, as observed in Table 7, generated in the different compositions, decrease in maximum stress according to the type and concentration of bioactive used, especially for chitosan films containing CEO. In this case, the natural bioactive can to act as plasticizers, reducing the interaction between the polymer chains and conferring film flexibility [131]. Such behavior corroborates the results observed by SEM.

Curatives for normal skin have maximum stress between 2.5 and 16 MPa [132]. All other compositions investigated are within the established and can be employed for such application. In general, chitosan films containing natural bioactives underwent large deformations prior to their rupture, especially the compositions containing CEO, CS/3CEO-F (105.1%), CS/1CEO-F (101.2%) in relation to the CS films (43.0%). The films were shown to be flexible and adherent both in visual inspection and in the tensile test. So, the films containing CEO are compatible for application as dressings since normal skin has an elongation at break of approximately 70% [133]. On the other hand, for the films containing MEO, the average values of elongation are below 70%. This is possibly due to the elongation property being evaluated in dry films, a condition potentially different from that used in the final application, since the films produced can absorb between 11 and 19 times their weight in water, acting as plasticizer in the polymer matrix, thus improving such property [134].

The incorporation of 1% of the MEO did not practically alter the modulus of elasticity of chitosan, changing from 35.1 MPa (CS-F) to 32.3 MPa (CS/1MEO-M) in relation to the other compositions. Contrary behavior was observed by incorporating 3% of the MEO (8.6 MPa), possibly suggesting greater mobility to the molecular segments of the CS/3MEO-M. All the other compositions showed very low Young’s modulus, varying from 5.4 to 8.6 MPa, compatible with the behavior of elastomeric materials as previously reported. This behavior, in addition to being influenced by bioactives, may be directly related to the lactic acid used in the preparation of films, which confers a gelatinous characteristic, and the presence of CEO can play an important role as plasticizer, improving the flexibility of the samples and decreasing their rigidity [135,136].

According to the literature, the elastic modulus of the skin varies between 4.6 and 20 MPa, and may vary depending on factors such as age, skin color, history of lesions and genetic factors [117,137]. For this purpose, all films produced here may be considered suitable for use as dressings. In general, chitosan and chitosan/essential oils membranes have sufficient and compatible tensile properties to be applied as dressings.

## 4. Conclusions

Film-forming emulsions and films were obtained by incorporating different concentrations of clove (CEO) and melaleuca (MEO) essential oils into chitosan (CS). The type and the concentrations essential oils affected their properties. Film-forming emulsions had translucent aspect with cloudy milky appearance and showed antimicrobial properties. The CEO had the highest inhibition against the three strains studied. As regards the films properties, the coloration of the films was affected by the type and concentration of bioactives used. At higher EOs concentration, the chitosan/CEO films showed an intense yellowish coloration while the chitosan/MEO films presented a slightly yellowish coloration, but in general, all chitosan/EOs films presented good transparency in visible light besides flexibility, mechanical resistance when touched, smaller thicknesses than the dermis, and higher wettability than chitosan films, in both distilled water and PBS. Tensile properties, including elongation at break, increased with EOs incorporation due to the lubricant characteristics of the EOs in addition to the interactions developed between chitosan and EOs, which were also confirmed by FTIR. The EOs droplets were well distributed along the surface of the films in SEM data. The results suggest that chitosan films incorporated with these essential oils could be employed for wound-healing applications. However, further studies are necessary for considering the antibacterial activity of the chitosan films.

## Figures and Tables

**Table 1:** Hydrogen potential of chitosan solution and chitosan/essential oils emulsions.

Sample	pH			
	1	2	3	Average
CS-S	2.60	2.62	2.65	2.62 A
CS/1CEO-E	2.62	2.56	2.61	2.59 A
CS/3CEO-E	2.55	2.60	2.60	2.58 A
CS/1MEO-E	2.63	2.60	2.61	2.61 A
CS/3MEO-E	2.63	2.63	2.56	2.61 A

**Table 2:** Antimicrobial activity of natural bioactive, chitosan solution and chitosan/essential oils emulsions against of *Staphylococcus aureus*, *Escherichia coli* and *Candida albicans*.

Sample	Zone of Inhibition (mm)		
	*Staphylococcus aureus* Gram-Positive	*Escherichia coli* Gram-Negative	*Candida albicans* Fungi
CEO	36.0	16.0	21.0
MEO	9.0	10.0	12.0
CS-S	8.5	7.0	7.5
CS/1CEO-E	0.0	7.0	8.0
CS/3CEO-E	9.0	8.0	7.0
CS/1MEO-E	6.0	9.0	7.0
CS/3MEO-E	0.0	8.0	9.0

**Table 3:** Qualitative evaluation of chitosan and chitosan/essential oils films.

Sample	Color	Homogeneity	Flexibility	Adhesion
CS-F	Transparent	High	High	High
CS/1CEO-F	Light yellow	Intermediary	Intermediary	Intermediary
CS/3CEO-F	Light yellow ^+^	Low	Intermediary	Intermediary
CS/1MEO-F	Lightly yellowish	Intermediary	Intermediary	Intermediary
CS/3MEO-F	Lightly yellowish ^+^	Low	Intermediary	Intermediary

**+** Indicative of greater intensity in color.

**Table 4:** Thickness values of the films of chitosan and chitosan/essential oils.

Sample	Thickness (mm)
CS-F	0.13 AB
CS/1CEO-F	0.14 AB
CS/3CEO-F	0.17 B
CS/1MEO-F	0.11 A
CS/3MEO-F	0.15 B

**Table 5:** Wettability values of the chitosan and chitosan/essential oils films.

Sample	Contact Angle (°)	
	Distilled Water (pH = 5.2)	PBS (pH = 7.2)
	Average	Average
CS-M	64.2 A	67.7 A
CS/1CEO-M	53.7 A	62.8 A
CS/3CEO-M	47.7 A	58.3 A
CS/1MEO-M	64.4 A	56.9 A
CS/3MEO-M	66.6 A	65.8 A

**Table 6:** Swelling values of the chitosan and chitosan/essential oils films.

Medium	Sample	Swelling (%)		
		1 h	2 h	3 h
	CS-F	-	-	-
DW * (pH 5.5)	CS/1CEO-F	678.57 A	611.97 A	971.04 A
	CS/3CEO-F	-	-	-
	CS/1MEO-F	2450.76 B	2448.22 B	3018.96 B
	CS/3MEO-F	742.41 A	961.21 A	1227.62 A
	CS-F	-	-	-
PBS (pH 6.4)	CS/1CEO-F	3370.20 B	886.87 A	1149.14 A
	CS/3CEO-F	3565.60 B	1364.64 A	1536.49 A
	CS/1MEO-F	5176.63 C	3469.14 B	4229.68 B
	CS/3MEO-F	1336.1 7A	999.91 A	1161.00 A

* DW = Distilled Water.

**Table 7:** Tensile properties of the chitosan and chitosan/essential oils films.

Sample	Maximum Stress (MPa)	Elongation at Break (%)	Young’s Modulus (MPa)
CS-F	20.3 ± 4.5	43.0 ± 7.9	35.1 ± 6.6
CS/1CEO-F	5.2 ± 0.9	101.2 ± 5.8	5.4 ± 0.9
CS/3CEO-F	6.6 ± 1.7	105.1 ± 7.9	5.8 ± 1.2
CS/1MEO-F	11.7 ± 1.3	61.1 ± 3.8	32.3 ± 2.2
CS/3MEO-F	7.3 ± 0.4	53.6 ± 6.3	8.6 ± 1.4
